# Are all forms of defence lost on islands? Persistence of a defensive mutualism in six island colonists from New Zealand

**DOI:** 10.1098/rsbl.2022.0425

**Published:** 2023-04-19

**Authors:** M. Biddick

**Affiliations:** Terrestrial Ecology Research Group, Technical University of Munich, Freising, Germany

**Keywords:** plant defence, phyllosphere, island syndrome, insular evolution, plant–mite mutualism, tri-trophic interaction

## Abstract

The loss of defence hypothesis posits that island colonizers experience a release from predation on the mainland and subsequently lose their defensive adaptations. However, while support for the hypothesis from direct defensive traits is abundant, far less is known about indirect defensive traits. Leaf domatia are cave-like structures produced on the underside of leaves that facilitate an indirect defensive interaction with predaceous and microbivorous mites. I tested the loss of defence hypothesis in six domatia-bearing taxa inhabiting New Zealand and its offshore islands. No support for the loss of defence hypothesis was found. Changes in domatia investment were instead associated with changes in leaf size—a trait that has been repeatedly observed to undergo rapid evolution on islands. Overall results suggest that not all types of defence are lost on islands.

## 1. Introduction

Island organisms often differ from their mainland counterparts in remarkable yet predictable ways. Collectively, these ecological, morphological and behavioural differences comprise what is known as the *‘island syndrome’* [1–3]. For example, plants often exhibit evolutionary shifts toward reduced self-incompatibility (Baker's law) [4], monocarpy [5] and secondary woodiness [6,7] after colonizing islands. One of the most remarkable trends comprising the island syndrome is the loss of defensive adaptations. The loss of defence hypothesis posits that island organisms lose defensive adaptations after colonizing isolated islands if they no longer provide a fitness benefit in the absence of mainland predators and pathogens [8]. For instance, many endemic island birds like the dodo of Mauritius exhibited no fear of mammalian predators—including humans (unfortunately to their detriment). In fact, almost half of the endemic avifauna of the New Zealand archipelago went extinct following the introduction of mammalian predators [9,10]. Similar behavioural shifts are seen in other prey taxa like insular foxes, wallabies and lizards [11,12].

Many island plants have also lost defensive adaptations [2,13,14]. While prey animals change in response to the absence of mainland predators in their new environment, plants change in response to absent mainland herbivores [15]. Leaf spines, for example, are effective physical deterrents of grazing ungulates like deer and cattle [16,17]. However, on the California Channel Islands where ungulates have historically been absent (until recent introductions), many endemic plants produce larger leaves, fewer spines, and less phenol compounds than their most closely related mainland sister taxa [18]. Another remarkable loss of defence is seen in *Pseudopanax crassifolius* (lancewood) from New Zealand. Lancewood saplings produce long, hardened leaves with lateral leaf spines that are advertised with aposematic coloration [19]. Thought to deter large (now extinct) browsing birds called *Moa* [20], these morphological adaptations have been lost in populations of *P. crassifolius* inhabiting the Chatham Islands some 800 km away (where *Moa* were absent) [21].

However, not all forms of plant defence are physical. Many plants have evolved to defend themselves by facilitating beneficial interactions with herbivore and pathogen enemies (i.e. indirect defence, [22–24]). Swollen-thorn acacias, for example, maintain an obligate mutualism with *Psuedomyrmex* ants by providing them with food (nectar and lipid-rich food bodies) and nesting sites (hollow thorns), which the ants pay for by protecting the trees from herbivores and competing plants [25–27]. Similar plant–ant mutualisms are observed in a variety of plants [28], such as *Cecropia* [29,30], *Piper* [31] and various bromeliads [32,33]. Facultative forms of indirect defence are also common (see [24] for comprehensive review).

**Figure 1.**
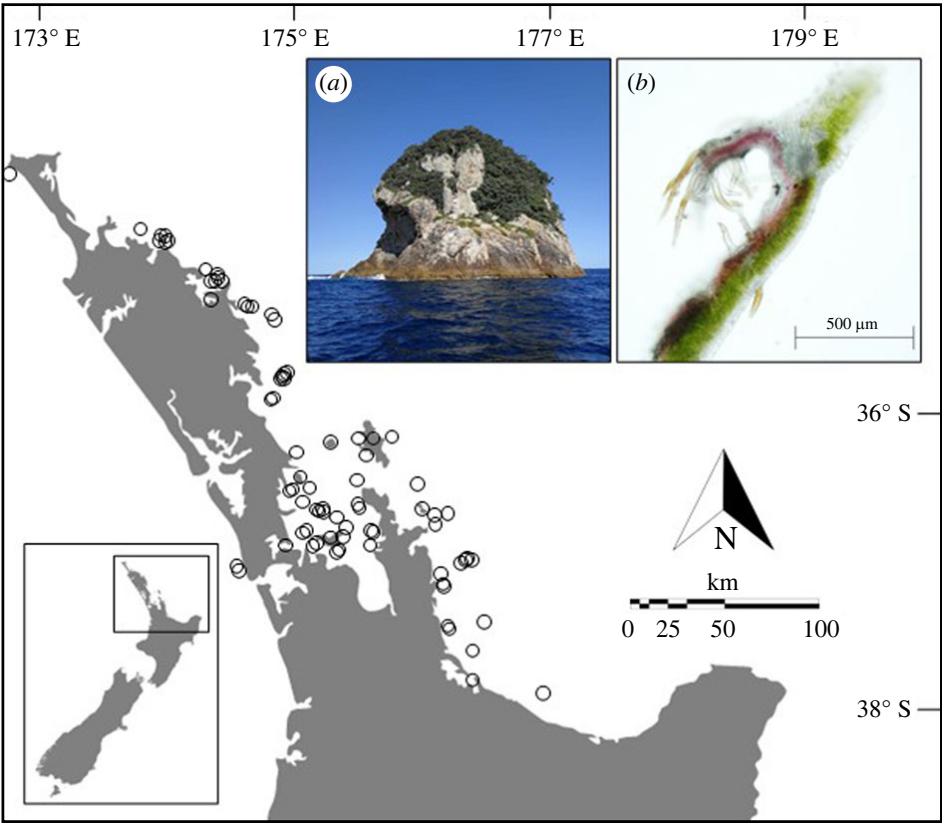
Study area. (*a*) Sixty remote islands off the east coast of the New Zealand ‘mainland’ were used to test the loss of defence hypothesis. (*b*) Leaf domatia are cave-like structures produced on the underside of leaves that facilitate a defensive mutualism with predacious and fungivorous mites (example of tuft domatia in *Carpodetus serratus* shown, photo credit: Morgan Ngata). Open circles denote individual offshore islands.

Some plants produce cave-like structures on the underside of their leaves known as domatia that house predatory and microbivorous mites [34,35]. Leaf domatia provide a refuge for oviposition and shelter from the environmental extremes of leaf surfaces, which mites in turn protect via the consumption of herbivorous arthropods and fungal pathogens [36–40]. Some 2000 species of domatia-bearing plants belonging to 227 plant families have been described [41]. Intriguingly, while domatia have been shown to be relatively uncommon in island floras [42], no study has documented whether leaf domatia are lost in island colonists. To test this hypothesis formally, I quantified domatia production in six cosmopolitan taxa inhabiting the New Zealand mainland and its surrounding offshore islands (figure 1).

## 2. Methods

### (a) Data collection

Data collection was conducted between June 2018 and April 2019. The six study taxa (*Coprosma repens* [Rubiaceae]*, C. rhamnoides, C. robusta, C. lucida, Elaeocarpus dentatus* [Elaeocapraceae] and *Vitex lucens* [Lamiaceae]) were chosen as they are the most widely distributed across New Zealand's north-eastern islands. All taxa produce pit domatia, except *E. dentatus* which produces tent domatia. Mainland field sampling was conducted in the Kaimai-Mamaku Forest Park in Tauranga (37°41′S, 175°45′E). This site was chosen because the Kaimai Ranges span a large latitudinal extent of the north-eastern corner of New Zealand, and therefore represent the probable source pool for many island populations. Island field sampling was conducted on seven islands: Cuvier, Great Mercury, Red Mercury, Otata, Ruamahuaiti, Ruamahuanui and Waiheke.

Individuals were chosen haphazardly while walking through easily accessible forest sections. Only fully expanded, mature leaves were measured (1–3 leaves per individual from at least 30 individuals per site per species). Leaf length was measured as the longest linear distance from the most proximal to the most distal point of the leaf lamina using a digital calliper. Leaf width was measured as the widest distance across the leaf lamina perpendicular to the leaf length measurement. Leaf area was calculated as the product of leaf length and leaf width. Although more accurate methods of estimating leaf area exist (e.g. leaf scanners, image recognition software or using *ad hoc* leaf shape correction factors [43]), leaf *×* width calculations sufficed for the purpose of this analysis as it is not concerned with among species differences in leaf size *per se*. Couched in other terms, any errors associated with leaf area estimates are consistent between island–mainland comparisons. In a prior study incorporating the same species I trialled *ad hoc* correction factors and found they provided little utility [44]. Further, a leaf scanner could not be used as most of the islands included in this study are protected by the Department of Conservation, who do not allow destructive sampling protocols. Domatia were counted systematically in a basipetal direction with the aid of a USB microscope (Toolcraft DigiMicro 2.0 Scale). It should be noted that while this study quantifies defence investment by domatia count, experimental work with grape cultivars has demonstrated that mite abundance on the phylloplane can scale with domatia size [40].

To expand upon data gathered in the field, pressed herbarium specimens from a further 53 islands and 58 mainland sites from the Auckland War Memorial Museum Herbarium (AK) were measured. The Herbarium houses an extensive collection of high-quality, preserved specimens of both indigenous and exotic plants spanning the full geographical extent of the New Zealand landmass—including its offshore islands. To standardize sampling across specimens, three leaves per specimen were measured using the same methodology outlined above. Leaves were chosen haphazardly, and care was taken to not damage specimens (i.e. gloves and minimal calliper–specimen contact). The final dataset comprised 1129 observations from 60 islands and 59 mainland sites.

### (b) Data analysis

To test for mainland–island differences in domatia production, while simultaneously accounting for leaf size and exploring differences in domatia–leaf size scaling, I performed a linear mixed effects models of number of domatia production (total count per leaf) against leaf area (cm^2^), insularity (island, mainland), their interaction and species identity. Both domatia count and leaf area were logarithm transformed to confirm to linearity. One was added to counts of domatia before transformation to avoid biologically nonsensical values (i.e. negative infinity). Location was included as a random effect permitting intercepts to vary among sites. Analysis and data visualization were conducted in R v. 4.2.2 [45] using the ‘lme4’, ‘lmerTest’ [46] and ‘tidyverse’ packages [47].

## 3. Results

**Figure 2.**
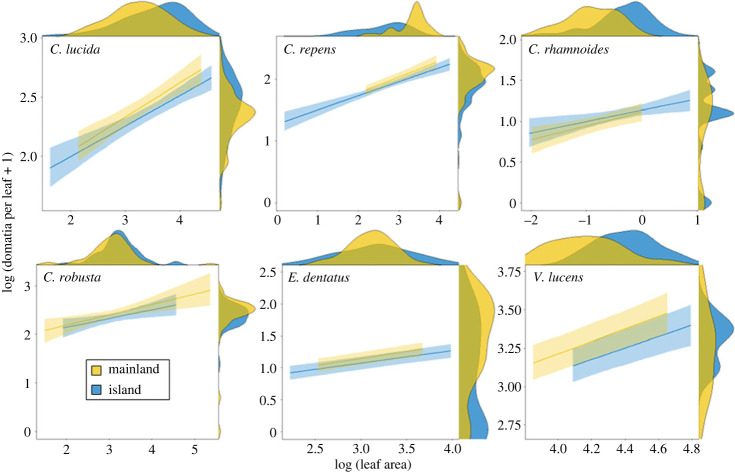
Leaf domatia production (*y*-axis, logarithm-transformed) and leaf area (*x*-axis, logarithm-transformed) in six cosmopolitan plant taxa inhabiting the New Zealand mainland (yellow) and its offshore islands (blue). Curves represent predictions from a reduced version of the model from table 1 without the random effect ‘location’ with 95% confidence intervals. Marginal density plots denote the distribution of observed data.

No consistent support for the loss of defence hypothesis was found (table 1, figure 2). After accounting for the significant scaling relationship between domatia and leaf size, island and mainland populations did not differ in domatia production, nor did the relationship between domatia production and leaf size change with insularity. Instead, changes in domatia production appeared to result passively from changes in leaf size due to them being allometrically linked. Only one species, *Elaeocarpus dentatus*, exhibited substantially reduced domatia production on islands that was not accompanied by a concomitant change in overall leaf size. It is worth noting that *E. dentatus* was the only species in this study that produces tent domatia and this may underpin its unique response to insularity. No species lost domatia entirely.

**Table 1.** Results of linear mixed effects model analysing the relationship between number of domatia per leaf (logarithm-transformed+1) and leaf area (cm^2^, logarithm-transformed), insularity (island, mainland), their interaction (i.e. changes in domatia–leaf size scaling), and species. Species-wise effects on domatia per leaf are in reference *C. lucida*. Location was included as a random effect allowing the intercept to vary among sites. Samples sizes (leaves) are denoted in parentheses for mainland and island populations, respectively. Significant *p*-values < 0.05 are indicated in bold. Number of observations: 1129. Number of groups (random effects location): 119.

parameter	estimate	std. error	*t*-value	*p*-value
fixed effects				
(intercept)	1.791	0.10	17.451	**<0.001**
leaf area	0.155	0.03	5.896	**<0.001**
insularity (m)	0.042	0.08	0.554	0.580
*C. repens*	−0.317	0.06	−5.807	**<0.001**
*C. rhamnoides*	−0.728	0.11	−6.575	**<0.001**
*C. robusta*	0.024	0.06	0.408	0.683
*E. dentatus*	−1.233	0.06	−19.560	**<0.001**
*V. lucens*	0.760	0.08	9.501	**<0.001**
leaf area : insularity	0.034	0.02	1.513	0.132
random effects	variance	std. dev.		
location (intercept)	0.055	0.235		
residual	0.158	0.398		

## 4. Discussion

Many previous studies have documented the loss of defence adaptations in island organisms, and loss of defence is considered more generally to constitute part of the ‘island syndrome’ [1–3]. However, findings from this study demonstrate that not all types of defence are lost on islands. Significant reduction in leaf domatia production on islands that could not be explained by concomitant changes in leaf size was only observed in one of the six study taxa. The remaining taxa either showed no change or, contrastingly, increased domatia production on islands.

Why are leaf domatia not lost on islands? The putative explanation for defence loss in island organisms is the absence of mainland enemies (predators and herbivores). For instance, mammals are typically poor dispersers and thereby lack the ability to colonize remote islands. Plants that have historically been defended against such mammalian herbivores, and subsequently colonize isolated islands, are freed from this herbivore pressure, and no longer benefit from the production of physical deterrents like thorns, spines and prickles. The mites inhabiting leaf domatia, on the other hand, protect plants against insect and fungal attack—both of which disperse readily to islands [48].

Another potential explanation is that the islands considered in this study, and the flora inhabiting them, are too young to exhibit the loss of defence phenomenon. Indeed, previous work has demonstrated the effect of island age and time since divergence on phylogeographic patterns [49,50]. Most of the islands considered here are continental break-offs from the larger New Zealand landmass. As such, they are relatively young in comparison to oceanic islands like Hawaii or Mauritius. This could explain why a marginal decrease in domatia production was observed in some taxa. Further, many islands are only weakly isolated from the New Zealand mainland, such that gene flow between island and mainland populations may exist, dampening the signal of an otherwise real effect (see [51] for analagous case).

Alternatively, the persistence of leaf domatia in island populations may be explained by their physiological cost (or lack thereof). While ant–plants like *Cecropia* incur a substantial expense to maintain healthy populations of their mutualist partners—primarily in the form of extrafloral nectar, protein-rich food bodies and hollow stem cavities that could otherwise be used for vascular tissue—plants with leaf domatia invest comparatively little to bolster populations of beneficial mites in the phyllosphere. As such, it remains plausible that leaf domatia persist in island taxa, not for reasons related to their ecological utility, but rather because the cost associated with their production is not substantial enough to be selected out of the gene pool. This may also explain why *E. dentatus*, the only species considered here with tent domatia, did exhibit reduced domatia production on islands. The relative physiological costs of different types of leaf domatia have yet to be quantified empirically, however results from this study suggest such investigation may be warranted.

Interestingly, while changes in domatia production were not associated with island colonization *per se*, they were instead associated with concomitant changes in leaf size. Prior work has demonstrated that leaf size in woody plants obeys the island rule [52]—a ubiquitous pattern in island evolution whereby species converge on intermediate sizes on islands. Changes in domatia production in island populations may therefore be explained by changes in leaf size as both traits are allometrically linked (though allometrically linked traits have been shown to sometimes evolve independently of one another [53]).

While this study found no support for the dissolution of the plant–mite mutualism on islands, there are clear conditions under which such mutualisms can be evolutionarily lost. For example, *Cecropia* plants inhabiting high altitude habitats of Costa Rica tend to stop producing food for their mutualist partner, *Azteca* ants, presumably because they do not occur above 2000–2200 m elevation there [29]. In this sense, mountain tops are analogous to islands in that they often harbour reduced or absent populations of mutualist partners, competitors and predators. I have observed a similar pattern in the genus *Coprosma*. Though leaf domatia are a key morphological trait used to identify members of the genus, they are rarely found on the leaves of alpine taxa—though this may be explained by their much-reduced leaf size [44]. Finally, it remains plausible that the island populations considered in this study were simply too young to exhibit what is an otherwise real phenomenon. After all, at biogeographic scales we see that leaf domatia are far less common in insular floras like New Zealand or the southern Korean islands than they are in broadleaf deciduous mainland floras [42].

In conclusion, island plants are not ‘poorly defended’ *per se*. Rather, they tend to lose adaptations to enemies that are absent on the isolated islands they colonize. Because insect and fungal attack are still very much a threat on islands, the indirect defensive interaction of plants with predaceous and fungivorous mites facilitated by leaf domatia may remain advantageous (or at least not deleterious enough to be lost evolutionarily). Nevertheless, future work should test whether the maintenance of leaf domatia (and indeed other indirect defensive traits) is observed in other island taxa. Finally, the loss of defence hypothesis should be amended to reflect *changes* in defence rather than its loss.

## Data Availability

Data and code are available from the Dryad Digital Repository: https://doi.org/10.5061/dryad.rbnzs7hg3 [54].
